# Comparative Nanofabrication of PLGA-Chitosan-PEG Systems Employing Microfluidics and Emulsification Solvent Evaporation Techniques

**DOI:** 10.3390/polym12091882

**Published:** 2020-08-21

**Authors:** Divesha Essa, Yahya E. Choonara, Pierre P. D. Kondiah, Viness Pillay

**Affiliations:** Wits Advanced Drug Delivery Platform Research Unit, Department of Pharmacy and Pharmacology, School of Therapeutic Sciences, Faculty of Health Sciences, University of the Witwatersrand, Johannesburg, 7 York Road, Parktown 2193, South Africa; divesha.essa1@wits.ac.za (D.E.); pierre.kondiah@wits.ac.za (P.P.D.K.)

**Keywords:** polymeric drug delivery, nanoparticles, biocompatibility, antifouling effect, microfluidics, emulsification solvent evaporation

## Abstract

Poor circulation stability and inadequate cell membrane penetration are significant impediments in the implementation of nanocarriers as delivery systems for therapeutic agents with low bioavailability. This research discusses the fabrication of a biocompatible poly(lactide-co-glycolide) (PLGA) based nanocarrier with cationic and hydrophilic surface properties provided by natural polymer chitosan and coating polymer polyethylene glycol (PEG) for the entrapment of the hydrophobic drug disulfiram. The traditional emulsification solvent evaporation method was compared to a microfluidics-based method of fabrication, with the optimisation of the parameters for each method, and the PEGylation densities on the experimental nanoparticle formulations were varied. The size and surface properties of the intermediates and products were characterised and compared by dynamic light scattering, scanning electron microscopy and X-ray diffraction, while the thermal properties were investigated using thermogravimetric analysis and differential scanning calorimetry. Results showed optimal particle properties with an intermediate PEG density and a positive surface charge for greater biocompatibility, with nanoparticle surface characteristics shielding physical interaction of the entrapped drug with the exterior. The formulations prepared using the microfluidic method displayed superior surface charge, entrapment and drug release properties. The final system shows potential as a component of a biocompatible nanocarrier for poorly soluble drugs.

## 1. Introduction

Nanostructured materials have demonstrated efficiency at improving bioavailability and reducing toxicity of bio-actives when implemented in the design of drug delivery systems [1]. Synthetic polymeric nanocarriers are the simplest delivery systems because of their facile preparation, their ability to efficiently incorporate bio-actives within their matrices, and thereafter provide controlled release either by erosion or diffusion mechanisms [1]. In particular, biodegradable poly(lactic-co-glycolic) acid (PLGA) has been commonly used. PLGA can be prepared via various routes [2] from the co-polymers glycolic acid and lactic acid, which is derived from green and renewable resources such as corn [3]. It is a material well suited to be implemented in the design of synthetic polymeric nanocarriers due to its malleable structure that can be optimized by tuning the ratios of its constituent co-polymers to suit various applications [4]. However, the hydrophobicity of PLGA activates the reticulo-endothelial system (RES), which is responsible for elimination of administered nanocarriers by opsonin proteins [5]. Hence a hydrophilic interface is critical in order to decrease this elimination (called opsonisation) [6].

Naturally occurring polymers such as chitosan can mimic the extracellular matrix of biological systems [7,8,9]. Chitosan is a cationic polymer, formed by the deacetylation of chitin obtained from the exoskeletons of crustaceans. When used as a coating in the design of nanosystems, it confers numerous benefits such as stealth, muco-adhesion, increased cell and tissue penetration, controlled drug release and increase in bioavailability and efficacy of the drug load [10]. Chitosan is biodegradable, biocompatible, non-immunogenic, and has displayed anti-tumoural, antimicrobial and antibacterial activity. It is the second most renewable biomaterial after cellulose [8] and is approved by the FDA for use in biomedical systems [9].

In the design of advanced nanocarriers there are generally two main regions of the nanoparticle structure that influences the biocompatibility, i.e., the nanoparticle core and the corona [11]. The core is considered to have no interaction with circulatory elements while the corona (typically 10 nm of the particle surface), exhibits properties that determines the response of the nanoparticles to the RES. RES-associated proteins (opsonins) bind efficiently to the surfaces of hydrophobic particles, and removes them from circulation, resulting in their elimination in the liver or spleen [11]. 

Studies have shown that this can be circumvented by coating such nanoparticles with chitosan [14,15]. Low molecular weight chitosan serves as a hydrophilic coating and provides a degree of stealth to PLGA nanoparticles [12]. A further increased in the degree of biocompatibility can be achieved by coating with polyethylene glycol (PEG) which is widely used for the surface modification of amphiphilic nanoparticles due to the strong hydrophilicity provided by the repeating glycol units within the PEG structural backbone [6]. Furthermore, previous studies have proven that PEG provides stealth properties by non-specific interactions with circulatory proteins [16,17,18].

**Figure 1:**
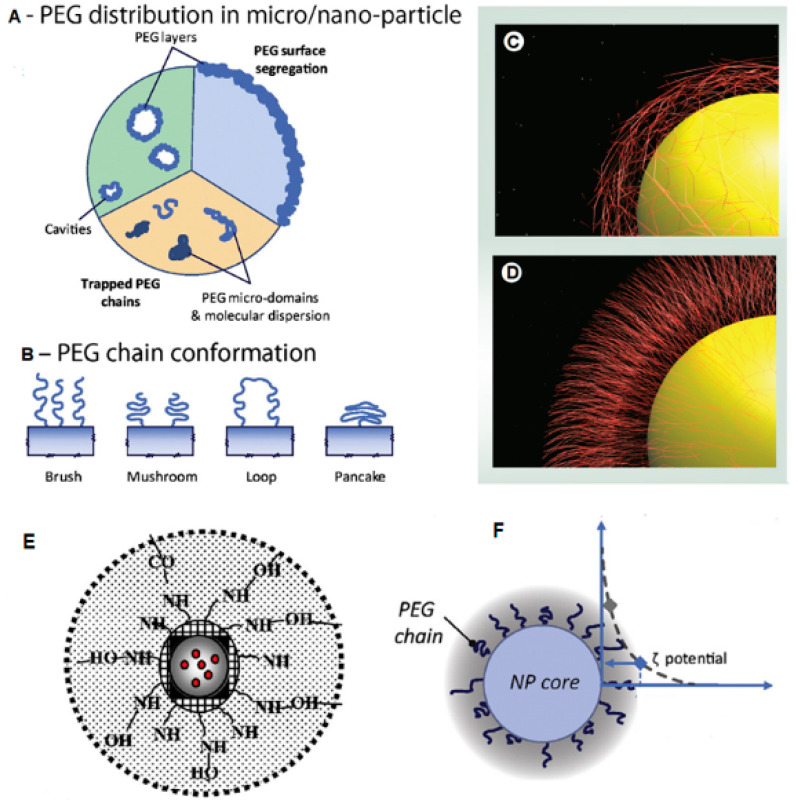
(**A**) Image representing different arrangements of PEG molecules in a nanoparticle. Adapted with permission from [12]. (**B**) Different conformations on nanoparticle surface. Representations of the mushroom (**C**) and brush (**D**) conformations, showing surface densities, reproduced from [11] with permission from Nanomedicine as agreed by Future Medicine Ltd. (**E**) schematic representation of the PLGA-chitosan-PEG nanoparticles prepared in this study. Reproduced with permission from [13]. (**F**) Nanoparticle surface showing detection of surface charge and PEG chains in both mushroom and brush conformations. Adapted with permission from [12].

PEG chains also introduce a degree of steric repulsion which further protects the nano-system from the RES [13]. Hereby, PEGylation provides an anti-fouling (or shielding) effect, and increases the nanoparticle circulation time. In passively targeted nano-systems for chemotherapy, this increased residence time can enable the system to access the enhanced permeation and retention (EPR) effect at the tumour site. The EPR effect takes advantage of the ‘leaky vasculature’ of tumour cells which have fenestrations >400 nm, allowing for preferential nanoparticle penetration with typically impaired tumoural lymphatic drainage resulting in nanoparticle accumulation within the cancerous cells rather than their escape back into circulation [6]. However, there is no current consensus on the optimal surface PEGylation density of nanoparticles that would induce and sustain favourable surface properties. Studies have shown that the structure of PEG occurs in two main conformations i.e., mushroom and brush [19] (Figure 1) with freedom of conformational mobility of PEG chains from attachment point on the core to the surface of nanoparticles in the brush conformation, while there is more lateral interaction in the mushroom configuration [6].

In order to fully exploit the individual properties of PLGA, chitosan and PEG for the design of an optimised nano-enabled drug delivery system, the particle size, dispersity, surface charge and structural organisation need to be precisely controlled. These characteristics are influenced in part by the preparation methods used, and it has been shown that the fabrication techniques can determine the overall functionality of these nano-systems [20], with the majority of PLGA-based nanoparticles synthesised via emulsion methods [21]. A less explored approach (but one with significant benefit) is the use of microfluidics for specialised nanoparticle synthesis [22]. Microfluidics is a relatively recently developed method that uses microchannel technology to blend mix phases under constant laminar flow, which cannot be performed using solvent emulsion. This approach has been previously reported to produce nanoparticles with more precise uniformity in particle size and dispersity and with a high degree of reproducibility [22]. In addition, microfluidics is a scalable technology [23] and therefore has promising applications in specialized drug delivery.

In this study, we compared two methods for the synthesis of PLGA-chitosan-PEG nanoparticles to encapsulate the model hydrophobic drug disulfiram (DSF) which is used clinically for the treatment of alcohol abuse [24] and, more recently, has been explored as a potential treatment for several types of cancer [25]. However, when tested as a chemotherapeutic, it has shown systemic instability [26] resulting in poor bioavailability. In this study, we prepared nano-systems incorporating PLGA as a hydrophobic core to encourage favourable interactions with disulfiram, cationic chitosan for preferential cellular penetration, and PEG as an outer layer for stealth properties. Microfluidics was used to provide reproducible nanoparticle synthesis with precisely controlled parameters (low variation) and was compared to a conventional emulsification method that typically displays large batch to batch variation [27]. In addition, previous PLGA-based nano-systems prepared using microfluidics have explored only one or two polymer components [28,29]. However, in this study a novel approach using a benchtop NanoAssemblr™ was used to attempt the synthesis using three polymers and a surfactant. The nanoparticles fabricated by both methods were characterised based on size, surface properties, thermal and molecular profiles.

## 2. Materials and Methods

### 2.1. Materials

Polymers, namely poly(d,l-lactide-co-glycolide) (PLGA) (lactide/glycolide ratio of 50:50, *M*_w_ = 7000–17,000 Da with acidic end groups, low molecular weight chitosan (CHT), polyvinyl alcohol (PVA) (*M*_w_ = 13,000–20,000 Da), disulfiram (model drug), dialysis tubing (MWCO 14 KDa), PBS buffer tablets (pH = 7.2), tween80 and mannitol were purchased from Sigma-Aldrich (St. Louis, MO, USA). Polyethylene glycol (PEG2000), glacial acetic acid, acetone, chloroform, dichloromethane, and acetonitrile were purchased from Merck (Pty) Ltd., (Estate South, Modderfontein, South Africa). All reagents were of analytical grade.

### 2.2. Preparation of Nanoparticles by the Emulsification Solvent Evaporation Approach

Various nanoparticle formulations were prepared using a single-emulsion oil in water (O/W) solvent evaporation approach [13]. Four initial formulation sets were prepared varying the solvent, surfactant concentration and sonication parameters, with an unmodified, unloaded PLGA formulation, an unmodified PLGA formulation loaded with disulfiram, a drug loaded PLGA formulation coated with chitosan and a drug loaded PLGA formulation coated with chitosan and PEG.

For each CHT-based formulation, 25 mg of CHT was agitated in 1% (*v*/*v*) glacial acetic acid to produce a 0.25% (*w*/*v*) concentration as the aqueous phase. The PEG-based formulations were prepared using 2% PEG and the PVA-based formulations were prepared using either 0.5%, 1% or 2% PVA. These constituted the aqueous phase and were prepared prior to the addition of the organic phase. The organic phase in each case was prepared by dissolving 100 mg of PLGA in 1 mL of either acetone, chloroform or dichloromethane and added dropwise under stirring to the aqueous phase. For the DSF-loaded formulations, 10 mg of DSF was added to the organic phase before addition to the aqueous phase. These concentrations and ratios were optimal for PLGA and DSF as previously reported [30]. The emulsion was left to agitate under magnetic stirring for 8 h to allow complete evaporation of the organic solvent.

For the sonicated formulations, the emulsion was sonicated for 2 min using a probe sonicator at 130 W with 40% amplitude. (Sonics Vibra Cell, Newtown, CT, USA) before the evaporation step. Thereafter the formulation was centrifuged (Universal 320, Hettich Lab Tech., Tuttlingen, Germany) for 30 min at 5000 rpm at 4 °C and washed thrice with distilled water to remove any unentrapped drug. The resultant pellets were redispersed in 4 mL of distilled water and then sonicated for 10 min to produce free-flowing nanoparticles that were frozen at −80 °C and then lyophilized (Freezone 12 lyophilizer, Labcono, Kansas City, MO, USA) for 24 h. A portion of sample of each formulation was mixed with 5% (*w*/*v*) mannitol to observe the effect of cryopreservation.

### 2.3. Preparation of Nanoparticles Using the Microfluidics Approach

For the microfluidics approach, polymeric nanoparticles were prepared using a benchtop Nanoassemblr (Precision Nanosystems Inc., Vancouver, BC, Canada). The defining characteristics of the nanoparticles produced by this approach were compared to those of the emulsification solvent evaporation method. The Nanoassemblr utilizes microchannels in a cartridge that allows for the mixing of organic and aqueous phase in precisely controlled speeds and ratios under laminar flow. The organic phase comprised 10 mg/mL PLGA and 1 mg/mL disulfiram dissolved in acetonitrile using the favourable ratio of polymer to drug (10:1) described earlier, and a concentration of organic phase polymer which was shown to produce relatively low particle size using this instrument [31].

Formulations were prepared using CHT (0.25% *w*/*v*), PEG (2% *w*/*v*) and PVA (2% *w*/*v*) in the aqueous phase. The particle size and stability were compared using flow rate ratios of 1:1, 1:3 and 1:5 and flow rates of 8, 12 and 15 mL min^−1^. Thereafter, DSF-loaded and native nanoparticles were prepared using CHT (0.25% *w*/*v*) and PEG (2% *w*/*v*) and loaded nanoparticles were prepared with 0.25% (*w*/*v*) chitosan only, and 0.25% (*w*/*v*) chitosan with 4%, 6% and 8% (*w*/*v*) PEG, using a flow rate of 8 mL min^−1^. The downstream processing protocol of centrifugation, washing, and lyophilization was followed as per the emulsification solvent evaporation method.

### 2.4. Determination of Particle Size and Zeta Potential

The nanoparticles were dissolved (1 mg of each formulation in 1 mL of Millipore water) and analysed using dynamic light scattering and photon correlation spectroscopy at a fixed angle of 90° on a ZetaSizer NanoZS (Malvern Instruments Ltd., Worcestershire, UK) particle size analyzer. Samples were diluted and sonicated before measurement, with the sample temperature maintained at 25 °C throughout the analyses. The particle size, polydispersity index and zeta potential values were used to screen formulations and select the most favourable preparation parameters such as the organic solvent, the concentration of stabilizer and sonication for the emulsification solvent evaporation approach. The flow rate ratio and total flow rate was used to screen formulations prepared by the microfluidics-based approach using the NanoAssemblr (Precision Nanosystems Inc., Vancouver, BC, Canada).

### 2.5. Determination of the Chemical and Structural Integrity of the Nano-Systems

The native polymers and different nanoparticle formulations were subjected to Fourier Transform Infra-Red (FTIR) analysis to assess the chemical integrity and stability of nanoparticles produced from the two approaches. Characteristic peaks were compared to confirm the molecular vibrations of the polymers in the formulations. The FTIR spectra were recorded using a PerkinElmer (Waltham, MA, USA) spectrometer equipped with a single reflection diamond MIRTGS detector. Samples were processed by a universal attenuated total reflectance (ATR) polarization accessory at a resolution of 4 cm^−1^ with a constant pressure of 110 psi. The chemical structures of each nanoparticle formulation prepared by either the emulsification solvent evaporation and microfluidic methods were compared.

### 2.6. Investigation of the Thermal Properties of the Nano-Systems

In order to characterize the thermal properties of the nanoparticles, thermogravimetric analysis (TGA) was undertaken using a TG analyser (TGA 4000, PerkinElmer, Llantrisant, Wales, UK), whereby the temperature range at which the formulation samples degraded were determined. Native DSF and DSF-loaded nanoparticle samples were exposed to 30 °C and then heated at a rate of 10 to 800 °C min^−1^. The samples were maintained in an inert environment by constant nitrogen purging throughout the experiment. Thermograms were plotted as percentage mass against temperature. The phase transitions of the fabricated loaded and blank nanoparticles and native polymers were investigated using a differential scanning calorimeter (DSC) (STAReSystem, Mettler Toledo, Schwerzenback, ZH, Switzerland). These analyses provide insight about the thermally induced phase changes that the samples undergo, and hence characterizes the variation in thermal and physical properties with change in the constituent materials of the samples. The nanoparticles and drug samples of ~5 mg were weighed into aluminium crucibles which were sealed and then heated over a temperature range of 0 to 300 °C, at a heating rate of 10 °C min^−1^. The samples were maintained in an inert N_2_ gas atmosphere. Calorimetry spectra were recorded as a function of heat flow against temperature.

### 2.7. Determination of the Crystallinity of the Nano-Systems

Lyophilized DSF-loaded samples and the native polymers were finely milled. PVA crystals were dissolved in distilled water and dried to form a film. Each sample was finely packed and smoothed into a sample holder for analysis using a benchtop MiniFlex 600 (Rigaku, Tokyo, Japan) powder X-ray diffractometer. CuKα radiation at 40 kV and 15 mA was employed and data was recorded using a 2θ scan range of 10–60° at a scan rate of 10° min^−1^. Powder X-ray diffractometry (XRD) analyses indicate the degree of crystallinity and amorphous nature of the polymers and nanoparticles and hence provides insight on their surface properties and behaviour.

### 2.8. Investigation of Surface Morphology Using Scanning Electron Microscopy (SEM)

Lyophilized samples were re-dispersed in distilled water and a drop of each sample was placed on aluminium stubs and dried for 48 h. Solid samples were mounted on double sided carbon tape. In order to induce electrical conduction, samples were coated in a vacuum with a fine layer of gold using a sputter coater. Each sample was then analysed on a SIGMA 03-39 field emission scanning electron microscope (Zeiss, Jena, Germany) at 5–15 kV acceleration voltage under an argon atmosphere.

### 2.9. UV Spectrophotometric Analysis to Determine Entrapment Efficiency of Disulfiram

Due to the hydrophobic nature of disulfiram a UV spectrophotometric method of analysis was adapted from [32]. Briefly, disulfiram standards were prepared using a ratio of 1:1 PBS:methanol, hereafter referred to as specialized buffer matrix (SBM). A linear calibration curve was constructed using the Nanophotometer UV/Vis spectrophotometer NP80 (Implen, Munchen, Germany). Two µL of sample was pipetted onto the nanophotometer head and the absorbance was read at 216 nm. A combination approach was developed to extract the drug into a medium that would allow for analysis. 5 mg of nanoparticles were added to 2 mL of SBM. The mixture was then stirred at 37 °C for 48 h using an orbital shaking incubator (LM-530-2, MRC Laboratory Instruments Ltd., Hahistradrut, Holon, Israel) at 50 rpm. The resultant solutions were centrifuged at 4500 rpm for 10 min and the supernatant analysed by UV spectrophotometry for disulfiram content. Entrapment efficiencies of each formulation was calculated from the absorbance and were based on the initial mass of disulfiram used in the fabrication method, as shown by Equations (1)–(3) below:

(1) Maximum drug content=mass of disulfiram added/(mass of disulfiram+mass of polymers)

(2) Actual drug content=mass of disulfiram detected /mass of nanoparticles analysed

(3) Entrapment efficiency=actual drug content/maximum drug content×100%

### 2.10. In Vitro Disulfiram Release Studies

In vitro release of DSF from the nanoparticle formulations were determined using a dialysis tubing method [30]. The tests were performed in PBS (pH = 7.2; 37 °C). For the comparative release study between nanoparticles produced from the emulsification solvent evaporation and microfluidic approaches, 5 mg of both formulations and pure DSF were suspended in 2 mL of PBS and transferred into the dialysis tubing immersed in 50 mL of outer medium containing 0.5% (*v*/*v*) Tween 80 and agitated for 72 h at 37 °C. At predetermined time intervals (15, 30 min as well as 1, 2, 4, 8, 12, 24, 48 and 72 h), 1 mL of the external release medium was withdrawn and replaced with an equal volume of drug-free medium to maintain sink conditions. Parameters were kept constant for all formulations assessed and samples were filtered prior to DSF analysis at 216 nm.

### 2.11. Statistical Analysis

Data for preparation and characterization of nanoparticles and drug release studies were processed and analyzed by Origin software (version 8.5.0 SR1, OriginLab Corporation, Northampton, MA, USA). All results were represented by the mean ± standard error of three experiments.

## 3. Results and Discussion

### 3.1. Size and Stability Analysis of Formulations Prepared by Both Methods

Even though the abnormal tumour vasculature allows the penetration of particles <400 nm in size [33], particles >200 nm have been shown to accumulate in the liver or spleen [34]. Therefore, both methods were used with the ultimate goal of fabricating nanoparticles <200 nm in size, with a polydispersity index (PDI) of ~0.2.

#### 3.1.1. Dichloromethane as the Solvent of Choice in the Emulsification Method

**Figure 2:**
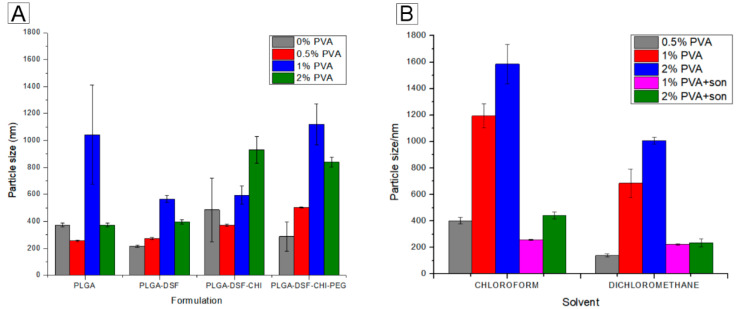
(**A**) Mean particle size obtained for formulations using acetone as the organic phase, and different concentrations of PVA in the aqueous phase. (**B**) Mean particle size obtained for formulations containing PLGA, chitosan and PEG with either chloroform or dichloromethane as the organic phase, different concentrations of PVA in the aqueous phase, and sonication.

Solvent evaporation using acetone in the presence of no stabilizing agent were the parameters initially used for the preparation of the nanoparticles. While this method produced nanoparticles in a desired size range for the unloaded and disulfiram loaded PLGA nanoparticles, as previously reported [30], the sizes and dispersity were erratic once the complexity of the formulations increased to include chitosan and PEG, and even when different concentrations of stabilizer were added, the size and dispersity did not improve, as shown by Figure 2 below, and Table S1 in the supplementary information. When chloroform, dichloromethane and sonication were used, the best formulation (with a size of 221 nm and a PDI of 0.028) was obtained with 1% (*w*/*v*) PVA, sonication time of 2 min, and dichloromethane as the organic phase, as shown in Figure 4. For a full list of results, refer to Table S1 in the Supplementary Information.

#### 3.1.2. Microfluidics-Prepared Formulations

The parameters were optimized using an aqueous phase of 2% (*w*/*v*) PVA, 2% (*w*/*v*) PEG and 0.25% (*w*/*v*) chitosan, and an organic phase of 10 mg/mL PLGA, with a PLGA:DSF ratio of 10:1 in acetonitrile. The best formulation (with a size of 218 nm and PDI of 0.165) was obtained using a flow rate ratio of 3:1 aqueous:organic phase and a total flow rate of 8 mL per minute, as shown by Table S2 in the supplementary information.

#### 3.1.3. Analysis of Emulsification Solvent Evaporation and Microfluidics Data Showing Dependence of Properties on PEG Surface Density

The emulsification solvent evaporation formulations were prepared using dichloromethane as a solvent, 1% (*w*/*v*) PVA and sonication time of 2 min. The microfluidic formulations were prepared using a flow rate of 3:1 and a total flow rate of 8 mL min^−1^. In this method, the mixing of the two phases were automated and controlled by the machine and hence the results obtained were highly reproducible. The uniformity of the dispersions was much better compared to the solvent emulsions method across all formulations as shown by the relatively low PDI values (0.191–0.258) and size disparity (218–330 nm) of the microfluidic preparations in Table S2, compared to the large variation in size and polydispersity of the solvent emulsion prepared formulations in Table S1. The microfluidics-prepared formulations also required fewer experiments before acceptable results were achieved.

Both emulsification solvent evaporation and microfluidic prepared formulations showed optimal properties when prepared with 6% (*w*/*v*) PEG in the aqueous phase, as shown by Table 1 and Figure S1A,B in the supplementary information. In the solvent emulsion method, the formulation with 4% (*w*/*v*) PEG displayed similar characteristics as that of the 6% (*w*/*v*) PEG. In formulations prepared by both methods, the uncoated PLGA nanoparticles have negative zeta potentials. The emulsification solvent evaporation uncoated nanoparticles display a larger negative surface charge, which is due to the larger relative amount of PLGA used in this formulation. When coated with chitosan, there is an increase in surface charge of both formulations, while there is an initial decrease when PEG (2% (*w*/*v*)) is added, then an increase until the PEG concentration reaches 6% (*w*/*v*), and a decrease once again when the PEG concentration is increased to 8% (*w*/*v*). The entrapment efficiencies of both formulations are high with PEG concentrations of 4 and 6% (*w*/*v*) and peak when the concentration of PEG is 6% (*w*/*v*). The entrapment efficiency of the PEG 6% formulations prepared by the microfluidics method (78.7%) was 8.4% higher than those of the solvent evaporation method (72.6%).

Acetone was not a suitable solvent for this system in this study, despite being the optimal solvent for a previously prepared disulfiram-incorporated PLGA-PEG system [30]. This could be due to the chitosan in the aqueous phase, which increased its viscosity, making it difficult for the emulsion to break into smaller droplets [35]. Furthermore, acetone is a water miscible solvent, hence it yielded particles with much larger sizes than dichloromethane, which is immiscible in water. This could be due to the faster rate of diffusion of acetone into the aqueous phase because of its miscibility, instead of the slow rate of diffusion which is required for the formation of smaller particles using the solvent evaporation method. The size and stability results are consistent with literature reports [35,36] which cite dichloromethane as a favourable solvent for PLGA nanoparticle formation by solvent evaporation. The formulations prepared by the microfluidics methods displayed a more uniform dispersity, as shown by Table 1, a more positive surface charge and greater entrapment efficiency than their solvent evaporation prepared counterparts. The more positive charge could be due in part to the larger amount of chitosan present in these formulations. The particle uniformity and stability could be due to the precise control of mixing rate and ratio that this method provides compared to the emulsification solvent evaporation solvent evaporation method.

The uncoated PLGA nanoparticles prepared by both methods displayed negative surface charges due to the carboxylic acid end groups on the PLGA. Chitosan becomes soluble in acidic media as the amine groups are protonated [7]; thereafter the hydrophilic polymer swells in aqueous solution, increasing the viscosity of the layer of water at the polymer surface, which explains the larger size of the nanoparticles coated only with chitosan. The positive zeta potential of the chitosan coated nanoparticles is due to the amino groups on the chitosan surface and its magnitude can be influenced by the surface area of the nanoparticles.

In theory, the larger the surface area, the more amino groups are present and therefore the more positive the zeta potential of the nanoparticle. The high surface charge of the nanoparticles also prevents particle aggregation in aqueous solution due to electrostatic repulsion [37]. The increase in zeta potential upon the coating of PEG may be due to multilayer deposition of PEG polymer during nanoparticle formation [13], which might cause interactions with the PLGA layer, exposing more of the cationic chitosan charge on the nanoparticle surface, as shown in Figure 1A. There are two major conformations of PEG on the surface of nanoparticles, with the brush conformation (present at higher PEG densities,) providing a higher degree of steric mobility than the mushroom conformation (found at lower PEG densities), as shown by Figure 1B. In this system, the optimal density of 6% (*w*/*v*) PEG resulted in particles with the highest entrapment efficiencies, suggesting a stabilizing effect on the amphiphilic structure, leading to a larger amount of hydrophobic drug-polymer interactions within the PLGA core.

The optimal entrapment efficiencies were similar for the formulations prepared by both methods, and this could be due to the optimal PLGA:disulfiram ratio of 10:1 [30,38] that was maintained in both methods, and demonstrates the dependence of drug entrapment properties on the compatibility of cargo with the core forming block [39].

Reports describe low PEG surface density in a mushroom conformation and as PEG density increases, the PEG chains reconfigure into a brush conformation [40,41]. With low PEG densities, the mushroom conformation allows free chains to move around on the chitosan surface and form multiple interactions, such as the intermolecular hydrogen bonding and electrostatic interactions between electropositive amine groups on chitosan and electronegative ether oxygen atoms on PEG [42]. This results in more shielding of the positive charge on chitosan, hence neutralising it to a large degree.

With higher concentrations of PEG, the conformations change from mushroom to brush, with steric repulsions between PEG chains causing reduced lateral interactions between PEG chains and chitosan surface, allowing for end chain PEG interactions only, as shown by Figure 1C,D. However, the conformational states of PEG on the nanoparticle surface can be complex due to multilayer depositions and interactions between these layers, as shown by Figure 1A. Therefore there could be a concentration of PEG close to the mushroom to brush transition density, that would form the optimal level of interactions between the polymers, utilizing the steric repulsion of the brush conformation and the topographical inhibition of protein binding provided by the mushroom conformation [19]. In the systems prepared in this study, this optimal concentration (6% PEG) provides the desired solubility and stealth effects due to the PEG shield, while the cationic properties of chitosan are still retained by the entire system, as shown by Table 1 and Figure 1F.

### 3.2. FTIR Spectral Analysis Showing Adsorption of Polymer Layers

**Figure 3:**
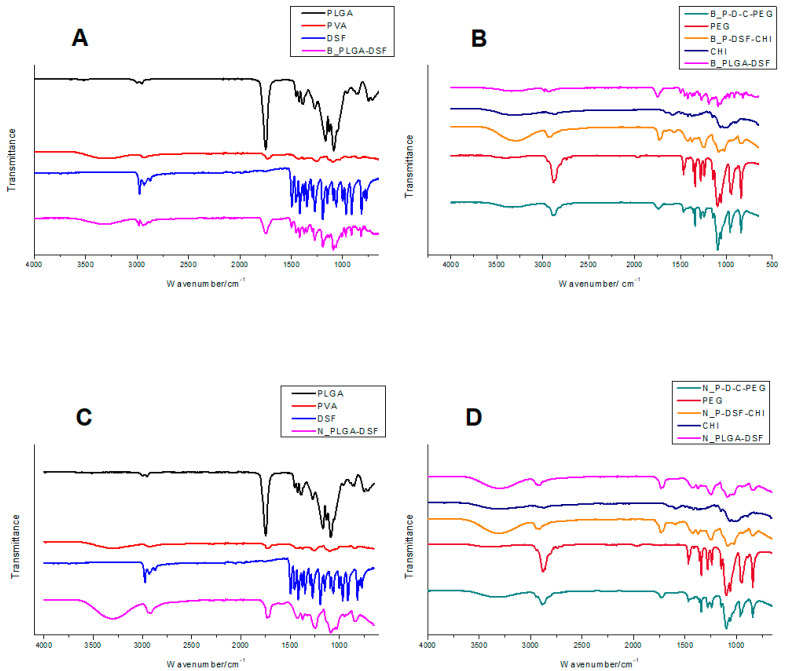
(**A**,**B**) FTIR spectra of the formulations prepared using solvent evaporation (bulk). (**C**,**D**) Formulations prepared using microfluidics (nano).

Figure 3 shows the spectra of the starting materials and formulations with increasing complexity prepared by both methods. The peaks in the PLGA spectra corresponded to bending of single carbon-hydrogen bonds at 500–1500 cm^−1^, stretching of carbonyl groups at 1775 cm^−1^, as well as stretching of single carbon-hydrogen (C–H), methylene (CH_2_), and methyl (CH_3_) bonds between 2885 cm^−1^ and 3000 cm^−1^. A methylene stretching peak due to ester bonds is also observed at 2955 cm^−1^ [36]. In the PVA spectrum, a broad peak due to hydroxyl groups is found at 3300 cm^−1^, a peak corresponding to methyl groups at 2800 cm^−1^, a carbonyl stretching peak at 1775 cm^−1^, and broad peaks due to C–H bending from 600 cm^−1^ to 1400 cm^−1^. The disulfiram spectrum shows a peak due to methyl stretching at 2975 cm^−1^, a peak at 1500 cm^−1^ due to C–H stretching, a peak at 1250 cm^−1^ due to C=S stretching and 1150–1200 cm^−1^ due to C–C stretching. Peaks at 950 cm^−1^ and 800 cm^−1^ were due to C–N and C–S stretching respectively, while peaks at 500–625 cm^−1^ were due to S–S bending [43]. In the chitosan spectrum, there is a broad peak at ~3400 cm^−1^ due to hydroxyl and amine stretching, and a characteristic amide peak at 1550 cm^−1^ [44]. In the PEG spectrum, a short, broad hydroxyl peak at 3250–3500 cm^−1^, a strong peak at 2800 cm^−1^ due to C–H stretching, and a characteristic peak at 1125 cm^−1^ can be observed due to C–O–C stretching vibrations [45].

The FTIR spectra show no new peaks on nanoparticles as coatings are added, and the spectra are superimpositions of the starting materials, with no peak shifts or new peaks, indicating no formation of new covalent bonds. This confirms that the coatings interact by physical adsorption only [44]. The nanoparticles prepared by both methods have very similar spectra, indicating that the manner in which the materials combine is the same for both methods.

### 3.3. Comparative Thermogravimetric Analysis and Differential Scanning Calorimetry of Disulfiram, Blank and Loaded Nanoparticles Prepared by Both Methods

**Figure 4:**
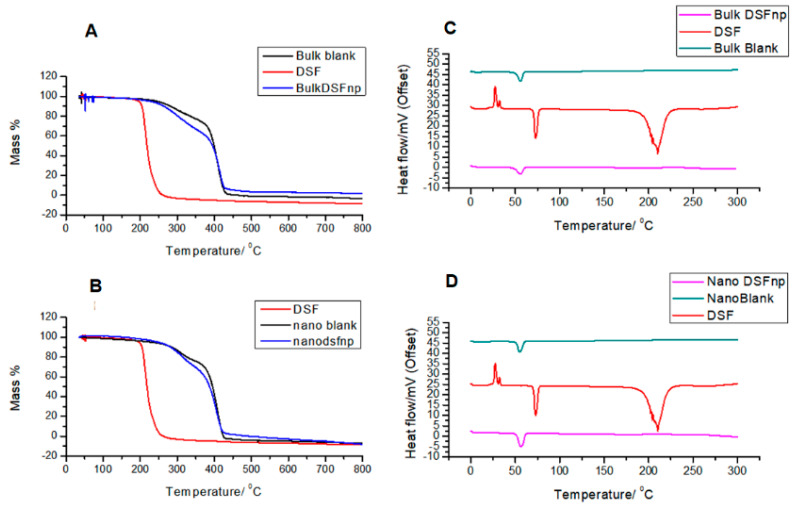
Graphs showing thermal degradation profiles of disulfiram, blank and loaded nanoparticles: (**A**) blank formulations. (**B**) microfluidic formulations. Relative DSC curves are shown with offset heat flow values for comparison: (**C**) emulsification solvent evaporation formulations. (**D**) microfluidic formulations.

The degradation of disulfiram occurred at 225 °C and degraded completely by 250 °C. The thermal degradation profiles of the formulations prepared by both methods were very similar. The blank nanoparticles prepared by both methods began degrading at 275 °C, with a slow degradation step until 410 °C, at which temperature 30% of the blank and 40% of the loaded nanoparticles had degraded, and a second step from 410 °C until complete degradation at 440 °C. There was still 8% of residue of the loaded nanoparticles by the end of the experiment. The loaded nanoparticles followed the degradation profile of the blank nanoparticles closely, as shown in Figure 4.

The thermogram for disulfiram show characteristic endotherms at 74 °C and 190–250 °C corresponding to melting and degradation events, respectively [46]. The thermograms for blank and loaded nanoparticles prepared by both methods were almost identical (Figure 4), with a characteristic endotherm for the glass transition temperature for PLGA nanoparticles shown at 55 °C [47].

The thermal and physical state of the nanoparticles influence its drug loading and release characteristics. The thermogravimetric analysis and differential scanning calorimetry data show that all the thermal events due to disulfiram were not displayed by any of the nanoparticle formulations, indicating that the loaded nanoparticles have adopted the thermal characteristics of the blank nanoparticles and that disulfiram is found physically dispersed in the nanoparticle system. The similarity of the formulations prepared by both methods indicate that they have similar physical states, which could be another reason for the similar entrapment efficiency observed in Table 1.

### 3.4. XRD Spectral Analysis Using Powder Samples of Pristine Polymers and Formulations Indicating Dominance of Blank Nanoparticle and PEG Surface Properties

**Figure 5:**
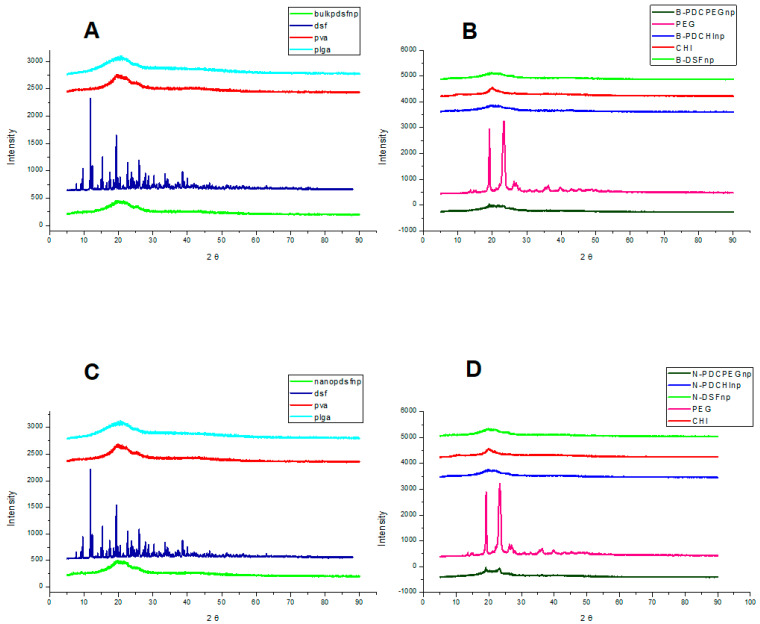
(**A**,**B**): X-ray diffraction spectra of the polymers and the loaded nanoparticles (2% PEG) prepared by the emulsification solvent evaporation method (bulk/B); (**C**,**D**): diffraction spectra of the microfluidic formulations (nano/N).

As shown by Figure 5, PLGA and PVA display amorphous surface characteristics while there are several crystalline peaks observed for disulfiram. The loaded nanoparticles show no crystalline peaks until coated by PEG. The PEG (2%) coated nanoparticles displayed small crystalline peaks at 2θ = 19° and 24°, which increased with increased amount of PEG coating, and this can be seen in Figure S2 in the supplementary information, which demonstrates the relatively larger crystalline peaks at 2θ = 19° and 24°, due to the higher concentration (6%) of PEG in these formulations.

XRD provides information of surface characteristics of nanoparticles, which is important for interactions within the circulatory system [48]. As illustrated by Figure 5, disulfiram is crystalline and its surface properties are not found when encapsulated in the nanoparticle formulations. The loaded nanoparticles’ surface is perceived in the same as that of the blank nanoparticles, suggesting that disulfiram is only present in an amorphous state within the nanoparticle. The surface properties show some crystalline peaks of PEG in Figure 5B,D, suggesting that PEG shielding should be in effect in these formulations.

### 3.5. Scanning Electron Microscopy of Lyophilized Samples Showing Spherical Morphology and Nanoparticle Adhesion in Microfluidics Prepared Formulations

**Figure 6:**
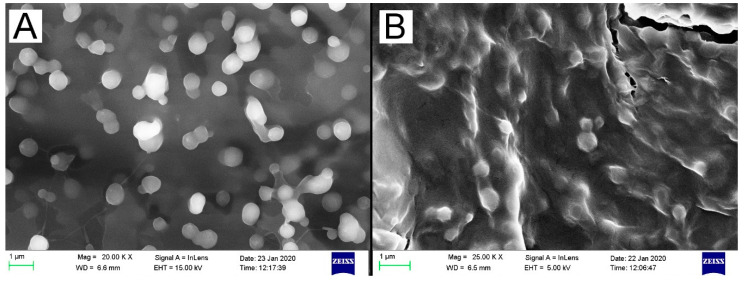
Scanning electron micrographs of nanoparticles prepared by emulsification solvent evaporation (**A**) and microfluidic (**B**) methods.

The use of mannitol to cryopreserve the samples had no effect except dilution and the samples that were evaporated on stubs were unstable and prone to cracking under the microscope. Hence all images were captured using solid lyophilized samples with no cryoprotectant. Figure 6 shows the spherical morphology of the nanoparticles prepared by both methods. There is a notable increase in nanoparticle adhesion observed in the particles prepared by the microfluidic method compared to those prepared by the emulsification solvent evaporation solvent evaporation method, as the former was prepared with a relatively larger amount of chitosan in the aqueous phase.

Both methods of preparation produced particles with spherical morphologies. The chitosan coating provides a positive charge which favours increased interaction with negative cell membranes. As previously reported [44], when the chitosan:PLGA ratio is greater than 0.4, there is a relative increase of chitosan on the surface of the nanoparticles which causes adhesion and this can be seen in the SEM micrographs of the nanoparticles prepared by the microfluidic method which had a chitosan:PLGA concentration of ~0.8, while the solvent evaporation method had a chitosan:PLGA concentration of 0.25. The degree of nanoparticle adhesion corresponds to the amount of chitosan present in the formulation and it has been demonstrated that higher ratios of chitosan:PLGA leads to increased cancer cell uptake of the nanoparticles which in turn results in higher cytotoxicity [44]. This result supports the relative zeta potentials of the particles prepared by the microfluidic method that were more positive than those prepared by solvent evaporation, which is another indication of the higher chitosan concentration.

### 3.6. Drug Release Kinetics of Disulfiram and Emulsification Solvent Evaporation and Microfluidic Formulations Prepared Showing Higher Relative Release of Microfluidics-Based Nanoparticles

**Figure 7:**
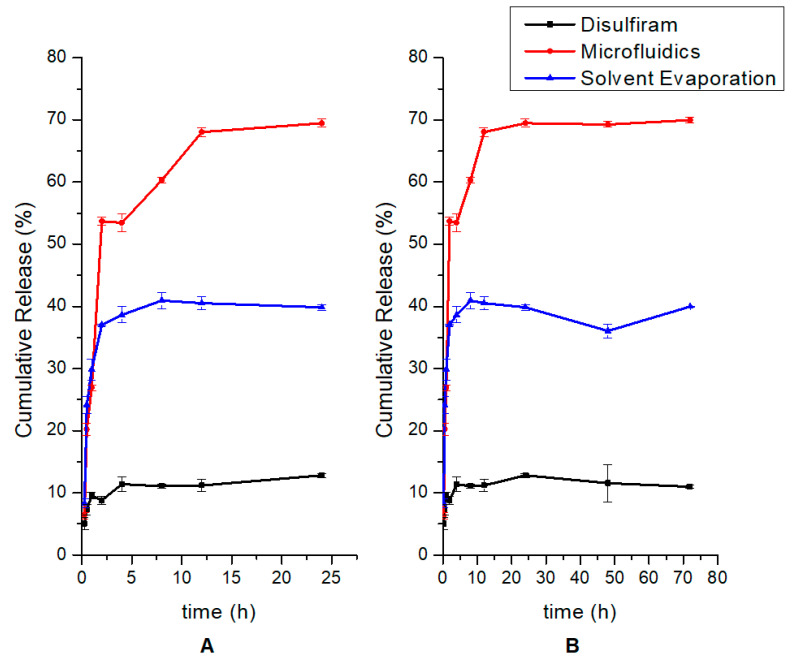
(**A**): 24 h and (**B**): 72 h cumulative in vitro release profiles of native disulfiram, emulsification solvent evaporation and microfluidic nanoparticles.

The drug release profile of the emulsification solvent evaporation and microfluidics prepared nanoparticles compared to native disulfiram is shown in Figure 7.

By 4 h, disulfiram had released 10% of its concentration into the buffer, with negligible release thereafter. Both nanoparticle formulations demonstrated biphasic release patterns, with initial burst release followed by sustained release. The nanoparticles prepared by the emulsification solvent evaporation and microfluidic methods released maxima of 40% by 8 h, and 70% by 12 h, of their disulfiram content, respectively.

Release behaviour is biphasic, with initial burst release caused by drug that is poorly bound by polymeric matrix or adsorbed on nanoparticle surface, while subsequent sustained release from PLGA core is further controlled by chitosan and PEG surface layers. PLGA degrades into its constituent co-polymers, lactic acid and glycolic acid, by hydrolysis of its ester linkages, while chitosan swells, providing a hydrogel-like layer which controls drug diffusion from the surface [37]. The profile of the microfluidic formulation displayed a slower release from 4 h to 12 h compared to the emulsification solvent evaporation prepared formulation. The dominant factor for the faster initial release of drug from the solvent emulsification preparation could be larger amount of drug adsorbed onto the surface of these particles, possibly due to larger surface pores of these types of particles compared to those prepared by microfluidics, as previously reported [49]. The microfluidic formulation also displayed a higher cumulative release over the 72 h period and a sustained release after 12 h. It has been demonstrated that microfluidics methods produce particles with a more uniform distribution of drug within the matrix compared to conventional emulsification methods [49], and this could be the reason for the more favourable release kinetics of the microfluidics preparation in Figure 7. The higher cumulative release of disulfiram from the microfluidic formulation could be due to a uniform structural organisation (compared to the solvent emulsion formulation) of the drug molecules within the polymer matrix, resulting in more reversible interactions, while the drug molecules could have been more tightly bound to the core in the solvent emulsion formulation, resulting in lower amounts of free drug released into the medium. It is worthy to note that although the entrapment efficiencies are in the same range for both formulations, the release characteristics of the microfluidic formulation displayed higher availability of the disulfiram content. The level of free disulfiram detected in solution was relatively low, which demonstrates its limited solubility in aqueous media that has been previously reported [50].

## 4. Conclusions and Future Work

We have designed and prepared polymeric nano-systems with a hydrophobic core, a cationic component and surface density of PEG chains that have resulted in favourable size, dispersity, surface charge and entrapment efficiencies. The hydrophobic PLGA core encouraged a high degree of carrier-drug interactions due to favourable drug: polymer ratio, while the chitosan and PEG coatings provided cationic and hydrophilic properties which are important for enhanced biocompatibility. The formulations prepared by solvent evaporation and microfluidics technique were physically, thermally and chemically similar. However, the difference in their physical surface properties was due in part to the difference in polymer concentrations which was an outcome of the method parameters. The formulations prepared by both methods entrapped the drug in an amorphous state, and did not retain any of its thermal or chemical properties, suggesting that the entrapped drug was efficiently shielded. The formulations also displayed cationic properties while maintaining the PEG groups on the nanoparticle surface. However, the microfluidics-based formulations displayed more desirable characteristics in terms of more positive surface charge which is associated with higher cancer cell uptake and toxicity, high entrapment efficiency with favourable drug binding and release properties, and higher cumulative and sustained release. These properties would make it highly effective for the delivery of drugs with low bioavailability, and could have applications for enhanced membrane permeation while retaining the greater benefits of antifouling effects in a passively targeted nanocarrier. Future directions of study with these formulations could include comparison of the hydrophilic properties that the different density of PEG shields convey to the delivery system, investigation of protein adsorption to the surface of the nanoparticles, and cytotoxicity and cellular uptake assays to investigate biocompatibility and therapeutic activity.

## Figures and Tables

**Table 1:** Size, dispersity, surface charge and entrapment efficiency of bulk and microfluidic prepared formulations with varying amounts of PEG coatings.

Formulation	CHI/%	PEG/%	PSD/nm	Polydispersity Index (PDI)	ζ Potential/mV	Entrapment Efficiency/%
Bulk	0	0	278 ± 1.0	0.100 ± 0.033	−17.2 ± 0.2	-
	0.25	0	245 ± 2.4	0.156 ± 0.000	14.0 ± 0.9	-
	0.25	2	221 ± 4.1	0.028 ± 0.020	3.09 ± 0.2	16.5 ± 1.0
	0.25	4	143 ± 3.7	0.183 ± 0.013	20.3 ± 0.9	72.1 ± 0.9
	0.25	6	184 ± 2.6	0.189 ± 0.011	21.3 ± 3.5	72.6 ± 0.2
	0.25	8	162 ± 10	0.155 ± 0.037	11.5 ± 1.4	26.5 ± 0.4
Microfluidic	0	0	95.8 ± 4.6	0.303 ± 0.031	−6.92 ± 0.7	-
	0.25	0	235 ± 1.9	0.292 ± 0.002	14.9 ± 1.0	-
	0.25	2	171 ± 14	0.284 ± 0.012	8.24 ± 1.8	29.9 ± 0.7
	0.25	4	220 ± 8.1	0.263 ± 0.001	19.2 ± 1.5	74.6 ± 1.0
	0.25	6	179 ± 2.5	0.247 ± 0.003	32.3 ± 0.1	78.7 ± 1.1
	0.25	8	204 ± 1.7	0.200 ± 0.006	18.3 ± 0.5	50.2 ± 0.2

## Supplementary Materials

The following are available online at https://www.mdpi.com/2073-4360/12/9/1882/s1: Table S1: Size and dispersity data for formulations prepared by the solvent evaporation method. Table S2: Mean particle size, dispersity and zeta potential data for formulations with varying flow rate ratios and total flow rates. Figure S1(A): Representative zeta potential plots showing surface charge variation with increasing % PEG coating density of solvent evaporation (bulk) prepared formulations; Figure S1(B): Representative zeta potential plots showing surface charge variation with increasing % PEG coating density of microfluidics (nano) prepared formulations. Figure S2: XRD spectra of the starting materials, blank and loaded nanoparticles (6% PEG) prepared by microfluidic (nano) and solvent evaporation (bulk) methods. Disulfiram is expressed as DSF.
