# Supplementary material for: Comparative Nanofabrication of PLGA-Chitosan-PEG Systems Employing Microfluidics and Emulsification Solvent Evaporation Techniques
Source: Polymers (Basel). 2020 Aug 21;12(9):1882. doi: 10.3390/polym12091882 (PMC7564778; doi:10.3390/polym12091882)
Supplement: Supplementary file 1 [file polymers-12-01882-s001.pdf]

polymers

MDPI

Supplementary Information

Comparative Nanofabrication of PLGA-Chitosan-PEG Systems Employing Microfluidics and Emulsification Solvent Evaporation Techniques

Divesha Essa, Yahya E. Choonara *, Pierre P. D. Kondiah and Viness Pillay *,†

Wits Advanced Drug Delivery Platform Research Unit, Department of Pharmacy and Pharmacology, School of Therapeutic Sciences, Faculty of Health Sciences, University of the Witwatersrand, Johannesburg, 7 York Road, Parktown 2193, South Africa; divesha.essa1@wits.ac.za (D.E.); pierre.kondiah@wits.ac.za (P.P.D.K.)

* Correspondence: Yahya.Choonara@wits.ac.za (Y.E.C.); Viness.Pillay@wits.ac.za (V.P.); Tel.: +27-11-717-2274 (V.P.)

† Professor Viness Pillay passed away shortly after the completion of the work

Supplementary Information

Table S1: Size and dispersity data for formulations prepared by the solvent evaporation method

Solvent	PVA /%	Formulation	PSD /nm	PDI
Acetone	0	Empty PLGA	373 ± 14	0,098 ± 0,05
		PLGA-DSF	216 ± 10	0,248 ± 0,05
		PLGA-CHI	485 ± 235	0,695 ± 0,1
		PLGA-CHI-PEG	288 ± 109	0,540 ± 0,02
	0.5	Empty PLGA	254 ± 5,9	0,423 ± 0,07
		PLGA-DSF	274 ± 8,9	0,139 ± 0,04
		PLGA-CHI	372 ± 8,1	0,176 ± 0,06
		PLGA-CHI-PEG	503 ± 5	0,074 ± 0,02
	1	Empty PLGA	1043 ± 368	0,736 ± 0,1
		PLGA-DSF	566 ± 26	0,298 ± 0,02
		PLGA-CHI	595 ± 68	0,485 ± 0,1
		PLGA-CHI-PEG	1119 ± 154	0,400 ± 0,03
	2	Empty PLGA	429 ± 117	0,635 ± 0,1
		PLGA-DSF	395 ± 15	0,257 ± 0,1
		PLGA-CHI	931 ± 97	0,827 ± 0,1
		PLGA-CHI-PEG	839 ± 36	0,728 ± 0,04
	0	PLGA-CHI(son)	792 ± 42	0,791 ± 0,02

		PLGA-CHI-PEG(son)	717 ± 21	0,454 ± 0,07
	0.5	PLGA-CHI(son)	271 ± 12	0,116 ± 0,07
		PLGA-CHI-PEG(son)	324 ± 19	0,354 ± 0,006
	1	PLGA-CHI(son)	372 ± 8,0	0,176 ± 0,06
		PLGA-CHI-PEG(son)	502 ± 4,9	0,074 ± 0,02
	2	PLGA-CHI(son)	808 ± 95	0,873 ± 0,1
		PLGA-CHI-PEG(son)	484 ± 23	0,660 ± 0,06
Chloroform	0.5	Empty PLGA	7914 ± 3812	0,653 ± 0,2
		PLGA-DSF	1369 ± 360	0,977 ± 0,02
		PLGA-CHI	1645 ± 157	0,993 ± 0,007
		PLGA-CHI-PEG	402 ± 26	0,391 ± 0,05
	1	Empty PLGA	1300 ± 333	0,985 ± 0,01
		PLGA-DSF	672 ± 16	0,709 ± 0,2
		PLGA-CHI	1294 ± 153	0,523 ± 0,2
		PLGA-CHI-PEG	1194 ± 91	0,252 ± 0,05
	2	Empty PLGA	1585 ± 149	0,246 ± 0,1
		PLGA-DSF	524 ± 114	0,576 ± 0,1
		PLGA-CHI	2059 ± 388	0,916 ± 0,1
		PLGA-CHI-PEG	868 ± 32	0,636 ± 0,3
	1	PLGA-CHI(son)	269 ± 9,8	0,169 ± 0,02
		PLGA-CHI-PEG(son)	257 ± 2,7	0,103 ± 0,02
	2	PLGA-CHI(son)	1097 ± 166	0,806 ± 0,1
		PLGA-CHI-PEG(son)	441 ± 26	0,123 ± 0,1
Dichloromethane	0.5	Empty PLGA	569 ± 42	0,765 ± 0,1
		PLGA-DSF	345 ± 14	0,397 ± 0,01
		PLGA-CHI	523 ± 130	0,407 ± 0,08
		PLGA-CHI-PEG	139 ± 12	0,989 ± 0,01
	1	Empty PLGA	10063 ± 6940	0,778 ± 0,1
		PLGA-DSF	2020 ± 241	0,244 ± 0,1
		PLGA-CHI	25316 ± 25316	1 ± 0
		PLGA-CHI-PEG	685 ± 106	0,656 ± 0,2

2	Empty PLGA	471 ± 245	0,529 ± 0,2
	PLGA-DSF	376 ± 44	0,416 ± 0,02
	PLGA-CHI	407 ± 20	0,368 ± 0,1
	PLGA-CHI-PEG	1006 ± 25	0,387 ± 0,1
1	PLGA-CHI(son)	844 ± 39	0,426 ± 0,2
	PLGA-CHI-PEG(son)	221 ± 4,1	0,028 ± 0,02
2	PLGA-CHI(son)	562 ± 55	0,476 ± 0,07
	PLGA-CHI-PEG(son)	234 ± 31	0,784 ± 0,1

Table S2: Mean particle size, dispersity and zeta potential data for formulations with varying flow rate ratios and total flow rates.

FRR	TFR /mLmin^{-1}	PSD /nm	PDI	Zpotential /mV
1:1	8	387 ± 6.3	0.191 ± 0.004	26.3 ± 1.3
1:1	12	238 ± 4.3	0.073 ± 0.004	24.2 ± 2.1
1:1	15	289 ± 2.3	0.066 ± 0.03	22.3 ± 2.2
3:1	8	218 ± 1.0	0.165 ± 0.01	34.5 ± 1.0
3:1	12	342 ± 2.0	0.127 ± 0.02	33.0 ± 1.4
3:1	15	304 ± 2.3	0.258 ± 0.002	28.5 ± 2.0
5:1	8	330 ± 9.8	0.126 ± 0.01	20.6 ± 0.6
5:1	12	248 ± 3.5	0.246 ± 0.007	25.7 ± 0.8
5:1	15	244 ± 6.0	0.246 ± 0.004	39.6 ± 3.8

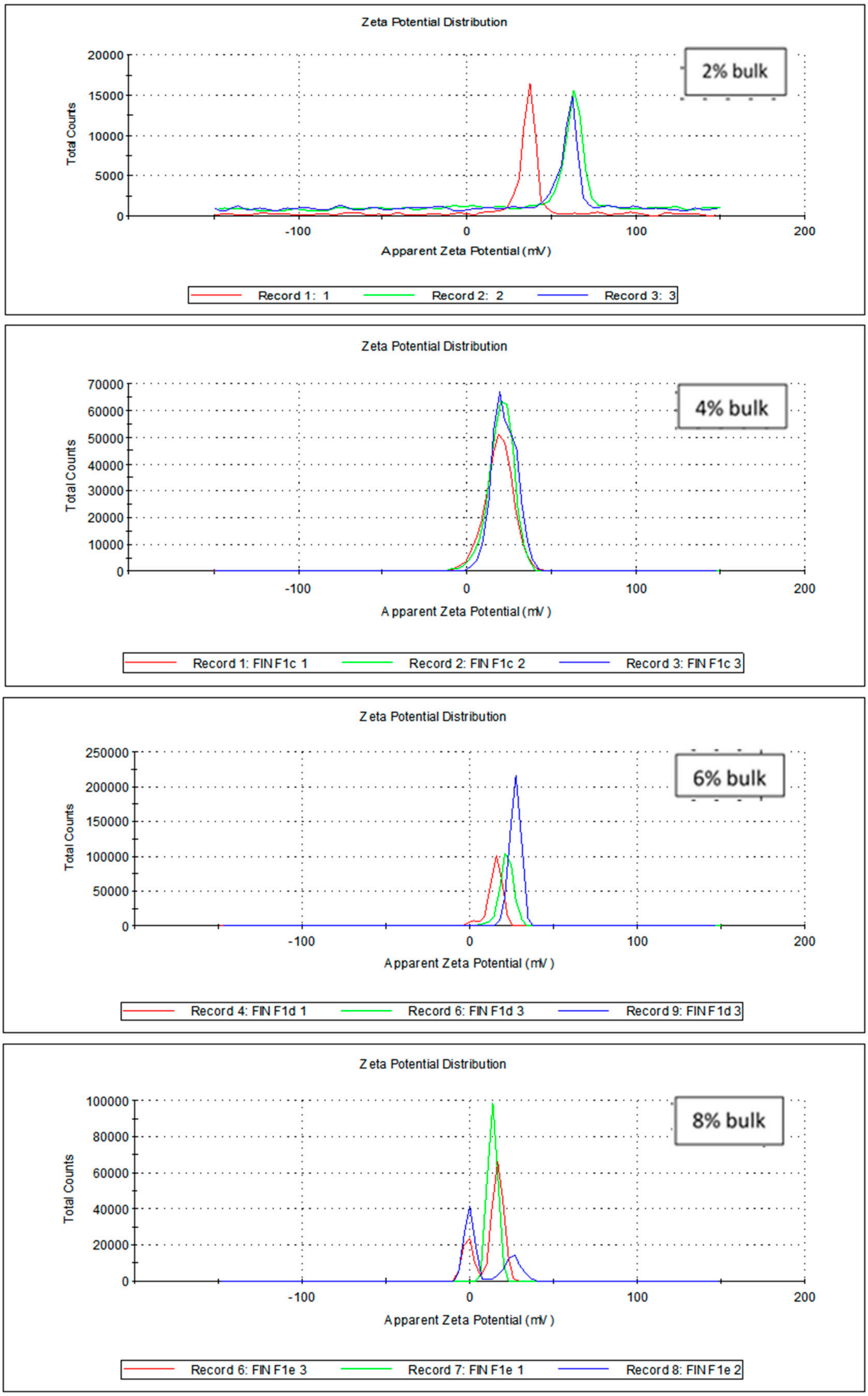

Figure S1(A): Representative zeta potential plots showing surface charge variation with increasing % PEG coating density of solvent evaporation (bulk) prepared formulations.

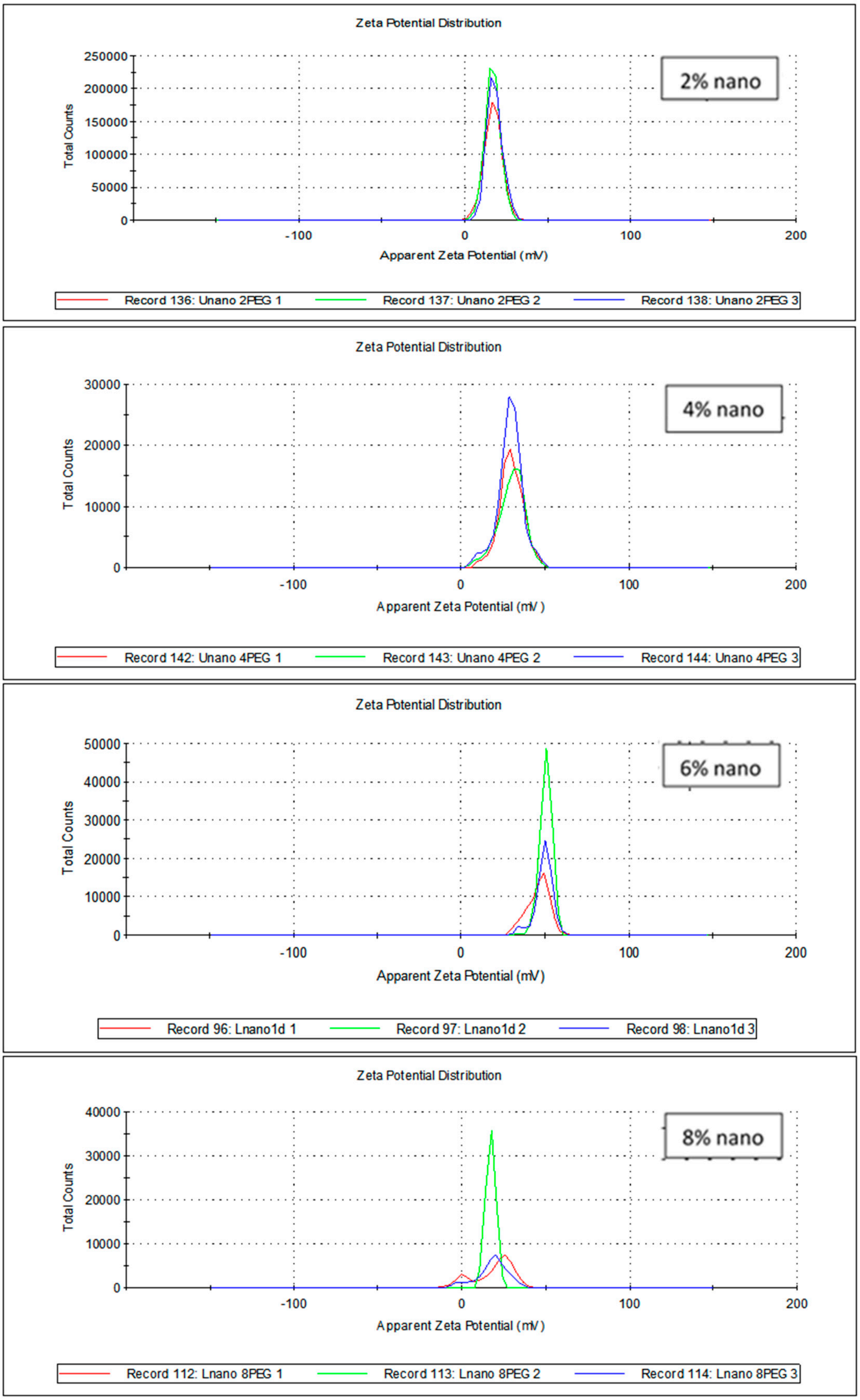

Figure S1(B): Representative zeta potential plots showing surface charge variation with increasing % PEG coating density of microfluidics (nano) prepared formulations.

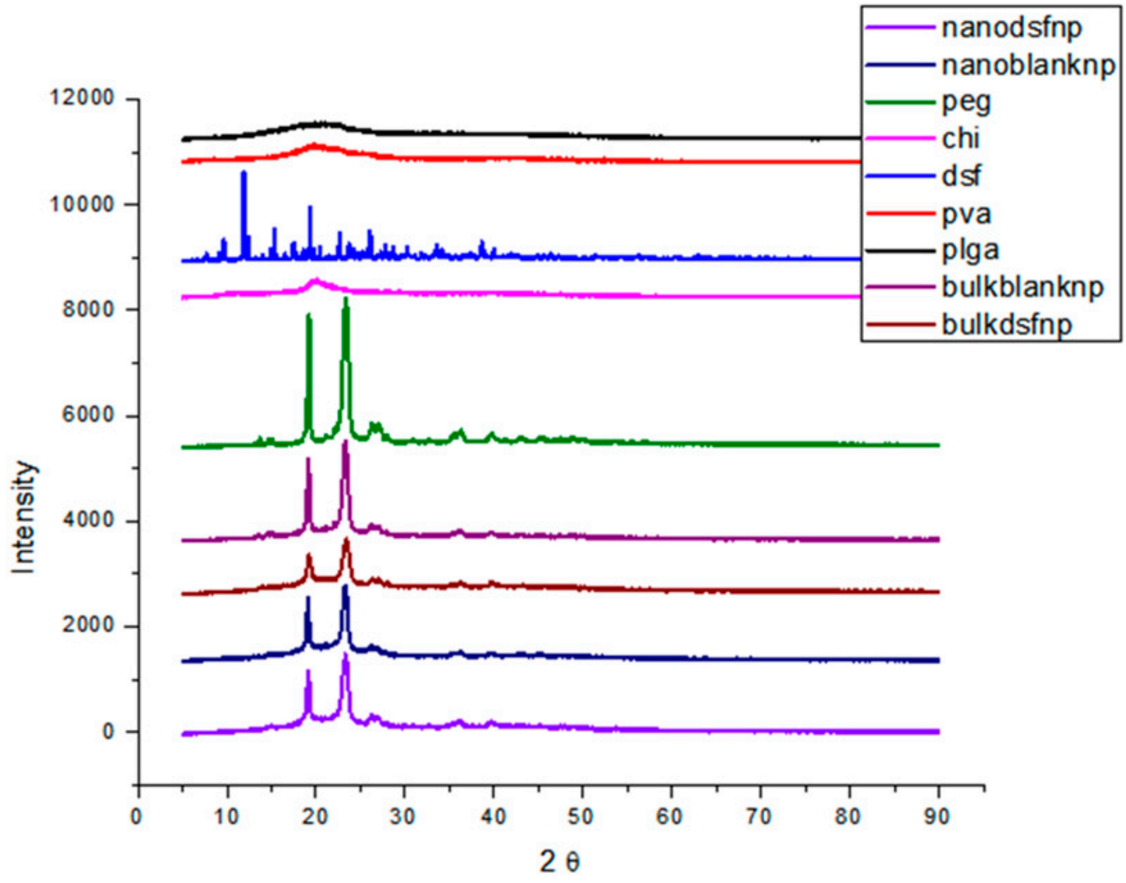

Figure S2: XRD spectra of the starting materials, blank and loaded nanoparticles (6% PEG) prepared by microfluidic (nano) and solvent evaporation (bulk) methods. Disulfiram is expressed as DSF.
